# Unveiling the Hidden Feast: A Model to Translate Molecular Detection Into Predation Rate—Application Example on Biological Control by Generalist Predators in Agricultural Fields

**DOI:** 10.1111/1755-0998.70033

**Published:** 2025-08-20

**Authors:** Abel Masson, Kévan Rastello, Ambre Sacco‐Martret de Préville, Yann Tricault, Sylvain Poggi, Elsa Canard, Marie‐Pierre Etienne, Manuel Plantegenest

**Affiliations:** ^1^ IGEPP, INRAE, Institut Agro, Univ Rennes Rennes France; ^2^ Department of Biology University of Victoria Victoria British Columbia Canada; ^3^ Functional Diversity in Agro‐Ecosystems Crop Research Institute Praha 6 Czech Republic; ^4^ IGEPP, INRAE, Institut Agro, Univ Rennes Angers France; ^5^ IGEPP, INRAE, Institut Agro, Univ Rennes Le Rheu France; ^6^ Crest, ENSAI Bruz France

**Keywords:** carabid beetle, hierarchical Bayesian model, molecular trophic ecology, pest regulation, predation rate

## Abstract

Few processes are as decisive as predation in shaping the structure and dynamics of ecological communities. For most predator species, however, the number of prey items killed by a predator in a day (predation rate) remains impossible to assess because direct observations are scarce or impossible to acquire. For such species, molecular gut content analyses are routinely used to test for the presence of a prey in the predator's gut. Specifically, our model uses a novel mechanistic representation of predation and digestion to integrate field data on prey detection and laboratory data on prey molecular signal decay in the predator's gut. Model fit provides an estimate of the slope and intercept of the digestion curve (molecular signal decay) and an estimate of the predation rate. In a case study targeting 25 carabid beetle species and 5 types of prey in agricultural fields (winter wheat), we use our model to estimate predation rates for each predator–prey pair. Based on predation rate estimates, we introduce a new biocontrol indicator at community scale and explore its potential for advanced agroecological research. We discuss the performance of our model on the basis of the scant information available in the literature and detail its conditions of application to highlight its advantages over existing predation models.

## 1 Introduction

The trophic ecology of predators shapes the network of interactions within predator–prey assemblages, playing a decisive role in ecosystem functioning and the provision of regulating services such as disease mitigation (O'Bryan et al. 2018) or pest control (Symondson et al. 2002; Bianchi et al. 2006; Snyder 2019). Prey populations are depleted by a diversity of predators at rates that depend on the diet of each predator species and its predation rate on each type of prey (see Supporting Information S2 for definitions). For some handpicked predators, the per capita predation rate on well‐known prey can be measured through direct observations in natural conditions (e.g., lions in the Kruger National Park (Mills and Shenk 1992) or birds foraging on intertidal preys (Wootton 1997)). For most species, however, predation can only be assessed indirectly. This is especially true for species that are difficult to observe, whether they are nocturnal, too small, living in remote or inaccessible areas such as telluric species, or because their behaviour is highly disrupted by observation. Terrestrial arthropod predators, for example, are ubiquitous but small animals involved in complex prey–predator assemblages in which direct observation of predation events is hardly possible. On the contrary, molecular approaches are well suited to small animals and commonly used to elucidate trophic links (Symondson et al. 2002; King et al. 2008; Traugott et al. 2013; Furlong 2015), but although these are two sides of the same coin, they are rarely used to estimate predation rates. We hypothesise that, despite recent developments (Andow and Paula 2023, 2024), a methodological breakthrough is still needed to quantify predation levels in complex predator–prey assemblages.

Apart from direct observation, two approaches can be used to obtain estimates of predation rates in real assemblages. A first approach is to parameterise the functional responses of predators to the density of their prey, typically with lab experiments or in field enclosure conditions (Pawar et al. 2012; Naranjo and Hagler 2001). But it comes up against two problems. Firstly, lab conditions often lack the complexity of natural environments, such as the presence of multiple prey species, environmental variability, or predator avoidance behaviours, which can significantly affect predation dynamics (Griffen 2021). Secondly, generalisation is difficult when trophic interactions are numerous but poorly understood, which is always the case in large assemblages of inconspicuous species. One way of reducing complexity could be to search for general relationships between the functional traits of predators and/or preys and the corresponding consumption rates, for example by predicting the predation rates using an allometric (body‐sized) relationship in a dynamic food web model (Curtsdotter et al. 2019). However, this promising approach requires high‐resolution data and raises technical and interpretive problems (Stell et al. 2024). Moreover, parameters are estimated by fitting the entire model to prey population dynamics data, with no possibility of comparing predation rates with other forms of measurement.

An alternative and straightforward approach to estimate predation rates relies on molecular analyses, which can detect prey remains in predator gut contents or faeces. Molecular data indicate either prey quantity or simple presence/absence, but can be encoded in multiple formats—including the proportion of predators testing positive or relative prey abundances in the remains. Historically, (Dempster 1960) introduced the idea of dividing the proportion of predators testing positive with prey detection time to provide rough estimates of predation rate. Following his idea, several more complex models were developed, integrating, for example, the number of preys consumed when tested positive (Rothschild 1966; Nakamura and Nakamura 1977; Greenstone 1979) or replacing digestion time with the half‐life of the prey's molecular signal in the predator as measured in the lab (Naranjo and Hagler 2001; Greenstone et al. 2010, 2014; Uiterwaal and DeLong 2024). Since the 1980s, other models (Lister et al. 1987) lay bare the assumption that, on average, the rate that prey are consumed equals the rate that they are eliminated (steady state assumption). Their approach consists of combining prey material decay rate and average prey material amount in field‐caught predators, and relies on quantitative methods such as quantitative electrophoresis (Lister et al. 1987), quantitative ELISA (Sunderland et al. 1987; Sopp et al. 1992) or qPCR (Andow and Paula 2023). Finally, the diet approach, notably used in marine ecology, aims to establish a relationship between prey proportions in the diet of the predator and in the detected remains. Diet models in the literature (Iverson et al. 2004; Coblentz et al. 2017) also rely on quantitative methods such as quantitative fatty acids signature analysis (Iverson et al. 2004) or Relative Read Abundance (Metabarcoding) (Deagle et al. 2019) and are best suited to cases where an exhaustive list of the predator's prey is available. However, quantitative methods remain less common than binary‐qualitative approaches, such as PCR (Furlong 2015; Pereira et al. 2023), mainly due to their higher cost and the fact that the associated uncertainties are still poorly characterised (Lamb et al. 2019). Since binary‐qualitative outputs are the most universal (all methods can ultimately yield binary‐qualitative information) and are already routinely used in many practical situations, we chose—following the example of Coblentz et al. (2017)—to develop a model that makes better use of binary‐qualitative data.

Like telescopes for astronomers, models can be used as observation tools to reach hidden information. In particular, models that are anchored in a faithful representation of biological systems allow inferring unobserved quantities through their observed effect on available data. Hierarchical Bayesian modelling (Ellison 2004; Fabre et al. 2010), that naturally accommodates latent variables, provides an accurate framework to develop such an observation tool. Indeed, HBMs allow integrating (i) a representation of all the biological processes of interest through the definition of latent variables, (ii) multiple sources of data relating to all or part of the system and (iii) all expertise on the system, encompassing both knowledge and uncertainty in the form of a priori distributions. To our knowledge, HBMs have never been used to estimate predation rates from molecular data. Yet they seem particularly well suited to the general problem of integrating two sources of data (laboratory and field data) and two processes (digestion and predation) to quantify an underlying variable (predation rate) (Ellison 2004). Furthermore, unlike frequentist approaches in the literature, they provide a simple and direct quantification of the uncertainties associated with the prediction (Cressie et al. 2009).

In this paper, we present a novel method that uses a hierarchical Bayesian modelling framework to provide estimates of in‐field predation rates based on two sources of molecular data: DNA‐based prey detection tests carried out on predators fed in the laboratory and on predators captured *in natura*. To illustrate the potential benefits of our method, we develop an example of its application to pest regulation by ground beetles (*Carabidae*) in wheat fields. To our knowledge, this is the first example of simultaneously quantifying predation rates of an entire predator community for such a large predator–prey assemblage of soil fauna.

## 2 Model Description

**FIGURE 1:**
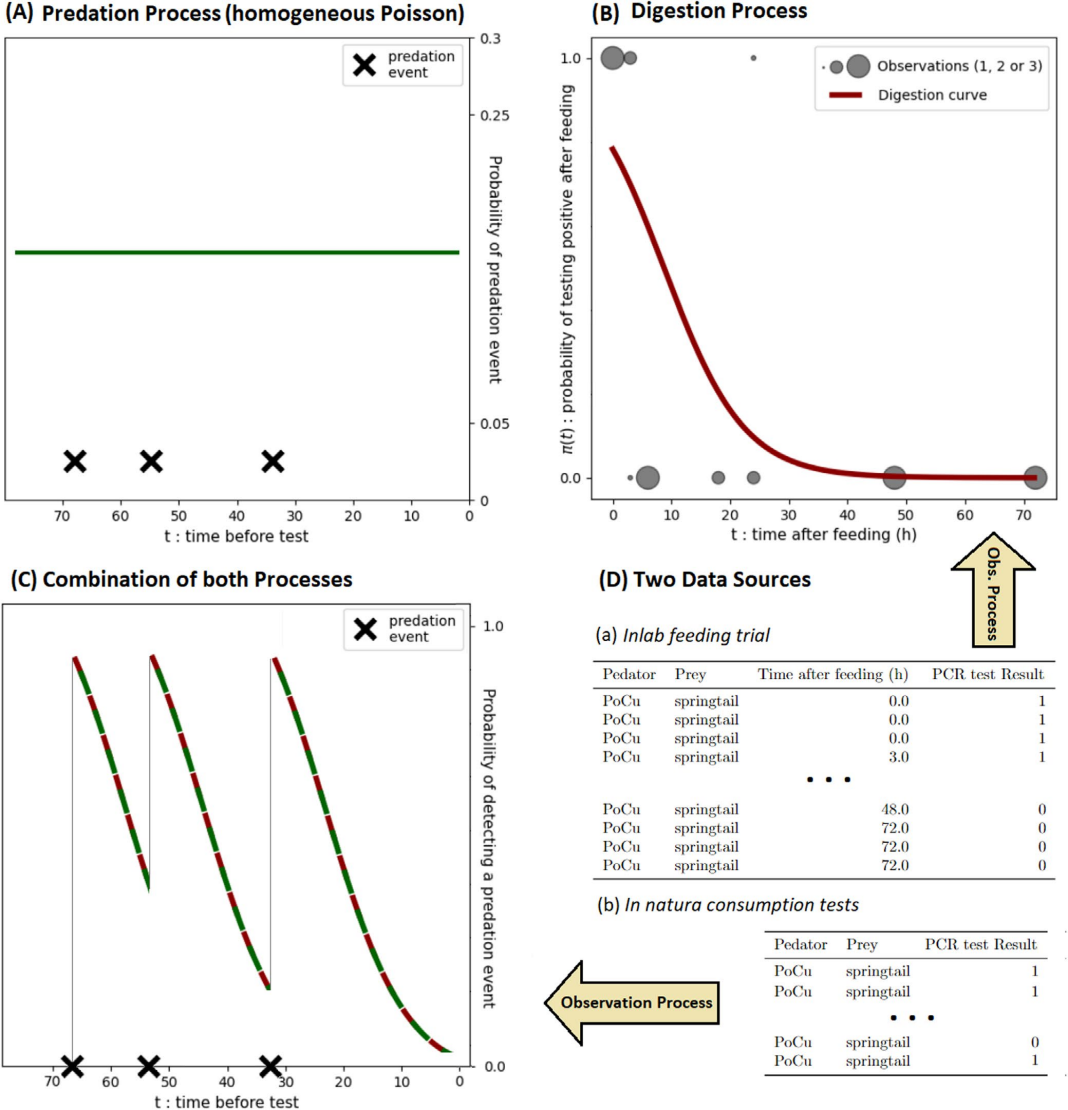
Schematic representation of the model framework. Panel (A) represents the predation process (in green) with constant predation rate and three predation events occurring in the 72 h preceding the PCR test. Panel (B) shows an example of a digestion curve (in red) compared to the observed data (in grey), which are worth either 1 or zero depending on whether the prey was detected or not (see ‘PCR test Result’ column of Table a in panel (D)). Finally, panel (C) represents the combination of predation and digestion (combination of red and green), showing the evolution of the probability of detecting a prey in a predator over time. The data in (D) are taken from an example of predation on springtails by the carabid predator Poecilus cupreus (PoCu), as presented in our application example (see Section 3.1).

Our approach is based on a hierarchical Bayesian model (HBM). All codes mentioned in this section are available in the Supporting Information. The modelling framework combines a process model, representing the consumption of a prey by a predator through a system of latent variables, and an observation model, which links the estimates of the latent variables to observed data. The process model accounts for a predation process (homogeneous Poisson Process) and a digestion process (exponential decay of prey material in the predator's gut (Greenstone et al. 2014)). We demonstrate that their combination is an inhomogeneous Poisson Process. It exploits information from two data sources: (i) laboratory data obtained during a feeding trial, consisting of feeding and testing predators at various times after feeding events to assess the decrease in prey DNA detection rate in their gut as a function of time (Greenstone et al. 2014) and (ii) prey DNA detection rate in field‐caught predators (Figure 1).

### 2.1 Digestion Process

Digestion degrades the prey's DNA, reducing the probability of detection. Let π be the probability of detecting prey's DNA in the gut content of the predator after a time t since predation. In line with (Greenstone et al. 2014), we assume that π decreases as a logistic function:

(1) $$\log_{\mathrm{it}}\left(\pi \left(t\right)\right)=\beta_{0}-\beta_{1}\cdot t$$

Throughout this document, we refer to the curve defined by π over time as the digestion curve. $\frac{\exp\left(\beta ₀\right)}{1+\exp\left(\beta ₀\right)}$ is the probability of detecting prey DNA just after consumption (at the start of the digestion experiment). *β*₁ (*β*₁ > 0) is the slope of the digestion curve, that is, the rate at which DNA detection vanishes. It should be noted that the voracity of the predator, that is, the quantity of prey consumed per meal, is not explicitly accounted for in our model, but depends implicitly on predator, prey and predator–prey interaction (see Section 2.4).

### 2.2 Predation Process

We assume that (i) predation events are independent (see Supporting Information S1 for violations of this assumption), (ii) the predator consumes only one prey item at each predation event and (iii) the probability of occurrence of a predation event in a short time interval is proportional to its length. Under these assumptions, the predation process is a homogeneous Poisson process, and $N_{t}$ the number of predation events during a time interval of length *t*, follows a Poisson distribution with parameter λt, where λ is a positive real parameter called the intensity of the Poisson process.

(2) $$\forall t\in R+,N_{t}\sim \text{Poisson}\left(\lambda \cdot t\right)$$

Here, the intensity λ is the average number of preys consumed per time unit (i.e., per hour), which we refer to as the predation rate.

### 2.3 Observation Processes

The first observation process links the expectation provided by the digestion model to the detection tests carried out during the feeding trial. The result of each test $T_{{\mathrm{lab}}_{d}}$follows a Bernoulli distribution with parameter $\pi \left(d\right)$, where *d* is the time elapsed since feeding:

(3) $$\forall d\in R+,T_{{\mathrm{lab}}_{d}}\sim \text{Bern}\left(\pi \left(d\right)\right)$$

Results of the in‐field detection tests also follow a Bernoulli distribution whose parameter is determined by the interactions between predation and digestion processes. A negative detection can occur either from an absence of predation, or because the test failed to detect predation, in particular when digestion has erased the DNA traces.

In (Supporting Information S3) we demonstrate that D, the (theoretical) number of preys that are detected at the time of the test follows a Poisson distribution $D\sim \text{Poisson}\left(\int_{0}^{\infty }\lambda \cdot \pi \left(t\right)\cdot dt\right)$. We also show that the integral $\int_{0}^{\infty }\lambda \cdot \pi \left(t\right)\cdot \mathrm{dt}$ converges and that $\int_{0}^{\infty }\lambda \cdot \pi \left(t\right)\cdot dt=\lambda \cdot \int_{0}^{\infty }\pi \left(t\right)\cdot dt=\lambda \cdot \frac{-\ln\left(1+\exp\left(\beta_{0}\right)\right)}{\beta_{1.}}$

Thus:

(4) $$D\sim \text{Poisson}\left(\mathrm{\lambda I}\right)\;\text{with}\;I=\frac{-\ln\left(1+\exp\left(\beta_{0}\right)\right)}{\beta_{1}}$$

Since we only need one prey to be detected for the test to be positive, the result of a field detection test $T_{\text{field}}$ follows a Bernoulli distribution of probability $\mathrm{pI}=P\left(D>0\right)=1-P\left(D=0\right)$.

Furthermore, from Equation (4) and the Poisson distribution formula, we have:

$$P\left(D=0\right)=e^{-\lambda \cdot I}$$

Consequently,

(5) $$T_{\text{field}}\sim \text{Bern}\left(\mathrm{pI}\right)\;\text{with}\;\mathrm{pI}=1-e^{-\lambda \cdot I}$$

### 2.4 Effects of Prey and Predator Types on Predation and Digestion Processes

#### 2.4.1 Parameter Decomposition

We assume that the decrease in detection rate with time since feeding depends on the prey, the predator and their combination (Greenstone et al. 2014).

For *β*_0_, we set:

$$\beta_{0}^{p,c}=\alpha_{\beta 0}^{p}+\delta_{\beta 0}^{c}+\gamma_{\beta 0}^{p,c}$$

With $\alpha_{\beta 0}^{p}$, the effect of prey type *p*, $\delta_{\beta 0}^{c}$, the effect of predator c and $\gamma_{\beta 0}^{p,c}$, the effect of the prey–predator pair (interaction effect). For the sake of simplicity, we did not distinguish different effects on $\beta_{1}$ and simply used a log‐normal distribution to ensure that the detection curve decreases with time since feeding ($\beta_{1}>0$in Equation (1)) (Supporting Information S4).

Similarly, the predation parameters were decomposed into a prey, a predator and prey–predator interaction effects as follows:

$$\ln\left(\lambda^{p,c}\right)=\alpha_{\lambda }^{p}+\delta_{\lambda }^{c}+\gamma_{\lambda }^{p,c}$$

The logarithm ensures that the predation rate $\lambda^{p,c}$ remains positive.

#### 2.4.2 Hierarchical Structure of the Model

**FIGURE 2:**
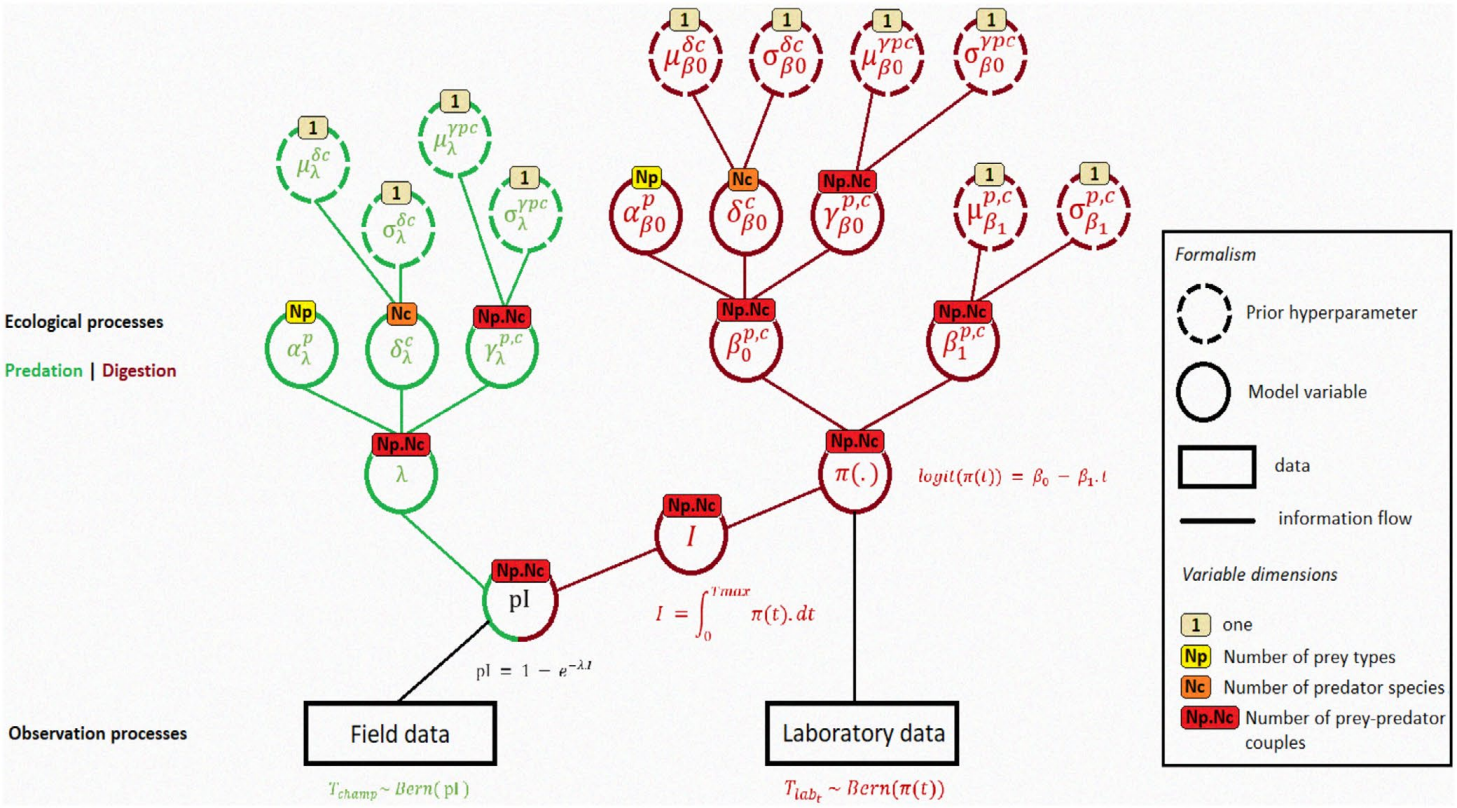
Full model specification—Hyperparameters (dotted circles) are those we used for our application example (see Supporting Information S4—prior specification). All abbreviations, parameters and variables are defined in Table SM 2.1 in Supporting Information S3.

In the case of strong functional or taxonomic similarities between predator or prey species, some of the prey, predator or predator–prey effects can be hierarchized, allowing, in particular, information to be transferred from well‐observed species to those for which observations are scarce. In our application, all predators belong to a single taxonomic group but the number of observations for predator and predator–prey effects varies greatly with many observations for some predator species and very few or even none for others. Hence, parameters $\delta_{\beta 0}^{c}$, $\delta_{\lambda }^{c}$ (predator effects), $\gamma_{\beta 0}^{p,c}$, $\gamma_{\lambda }^{p,c}$and $\beta_{1}^{p,c}$ (predator–prey interaction effects) are drawn from normal distributions whose hyperparameters, defined at the level of the entire taxonomic group, indicate the range of their plausible values. On the other hand, as there is no rare prey in the dataset and no taxonomic homogeneity between them, the prey effects are not hierarchized and are assessed independently. The complete structure of the model is shown on Figure 2 below.

## 3 Application Example

To illustrate the use of the model, we have applied it to the study of pest regulation by generalist predators, the ground beetles (*Carabidae*), in French wheat fields (Sacco‐Martret de Préville et al. 2022). We focused on the consumption of five types of prey: two groups of pests (slugs and aphids), two groups of decomposers (earthworms and springtails) and one group of natural enemies (spiders, reflecting intraguild predation). As the structure (composition and abundance) of carabid communities is highly seasonal, we used data collected over four sampling sessions (see Supporting Information S5). This example shows how our model can be used to estimate the predation rate at the predator species level (see Section 4.3) and how these results can be integrated at the community level (see Sections 3.2 and 4.4) to provide a new aggregate biocontrol indicator. We also show how our results compare with estimates of predation rates obtained using previously proposed methods (see Supporting Information S12).

### 3.1 Data

Two sources of molecular data have been used: one from laboratory feeding experiments and the other from field‐caught carabid beetles. Both datasets consist of a set of multiplex PCR results that indicate the presence/absence of the 5 types of prey DNA in the predator's gut. A total of 542 adults for the feeding trials and 1534 for the field captures belonging to 25 species of carabid beetles were considered in this study. Field‐caught carabids have been collected over 4 sampling sessions in 2018 and 2019 (Autumn (November & December), April, May and June) and in 4 regions of France (Île‐de‐France, Bretagne, Pays de la Loire and Lorraine). In the remainder of this article, ‘Autumn’ refers specifically to the months of November and December. For further details on the molecular method used (DNA extraction, primer design, multiplex PCR assay), and on data acquisition, please refer to (Sacco‐Martret de Préville et al. 2024).

Since (Sacco‐Martret de Préville et al. 2024) found that the capture session was the main driver of variation in the structure and activity of carabid communities in the study geographical range, we grouped the individuals caught during the same session in all regions to define 4 seasonal communities.

### 3.2 Biocontrol Indicator at Community Scale

We introduce a new community‐scaled biocontrol indicator as the sum of individual predator contributions to the regulation of each prey:

(6) $$\lambda_{\mathrm{com},p}=\sum_{c=1}^{N_{\mathrm{sp}}}\lambda_{c,p}A_{c}$$

With $N_{\mathrm{sp}}$ the number of carabid species in the community com, $\lambda_{c,p}$the predation rate exerted by carabid species *c* on the considered prey *p*, and $A_{c}$the abundance (or activity‐density) of species *c* in the community.

This biocontrol indicator was calculated for ground beetle communities of Autumn, April, May and June using abundances from each seasonal community but considering constant specific predation rates over the year. For each of the four seasonal community structures, the mean and variance of the indicator were estimated by calculating it with 100 sets of ${\left(\lambda_{c,p}\right)}_{c\in \left\{1,N_{\mathrm{sp}}\right\}}$drawn from their joint posterior distributions. For comparison purposes, rough estimates have also been calculated as in (Sacco‐Martret de Préville et al. 2024) by replacing the predation rate in Equation (6) with the proportion of positive detection tests in the observed data.

## 4 Results

### 4.1 Model Fitting

**FIGURE 3:**
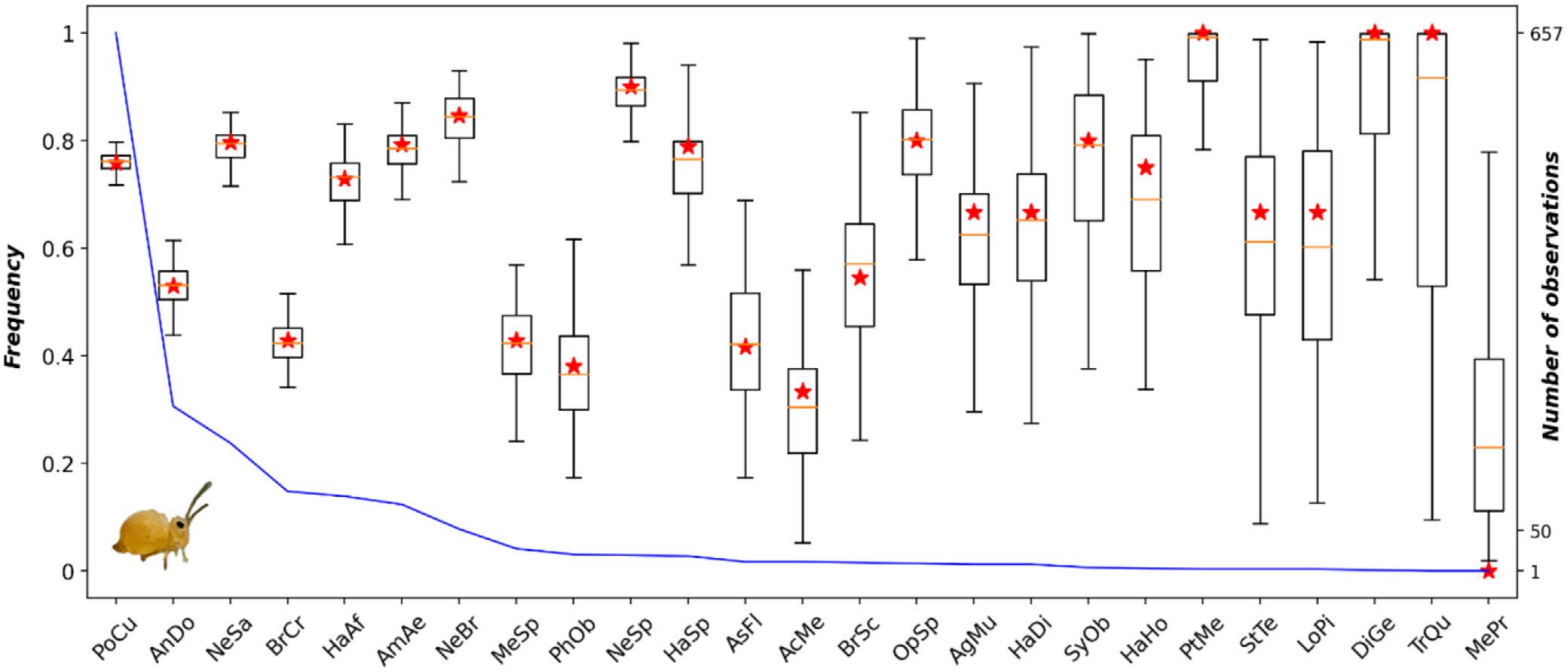
Goodness‐of‐fit: Posterior distribution of the detection probabilities compared to the observed frequencies of positive tests for each carabid species—Example of springtail detection. Posterior distributions of detection probabilities (pI) are summarised by the boxes, and observed values are indicated by red stars. Carabid species are listed from left to right in descending order of the number of individuals caught in the field (blue curve) ranging from 657 for Poecilus cupreus (PoCu) to 1 observation for Metallina properans (MePr), for a total of 1534 individuals (see Supporting Information S10 for carabid code names). The same figures for the other four prey types can be found in Supporting Information S9.

Using the priors specified in (Supporting Information S4), our model successfully estimates predation parameters for an assemblage of 25 carabid species (i.e., all species present at least once in both feeding experiment and field datasets) and 5 prey types (see Table SM 5.1 in Supporting Information S6 for mean values and standard deviations of daily predation rates). Convergence of the three Markov chains is validated by Gelman indices values (Gelman and Rubin 1992), all of which are less than 1.01, and visual examination of trace plots (see Figure SM 6.1 in Supporting Information S7) and posterior distributions (Figure SM 7.1 in Supporting Information S8). Figure 3 illustrates the goodness‐of‐fit of the model for field detection of springtails (see Figures SM 8.1 to 8.4 in Supporting Information S9 for the other prey). All observed frequencies of detection fall within the 95% interval of the posterior distribution of the detection probabilities (pI), and 90% of them stand between the 25th and 75th quantiles. The variability of estimates for each carabid species increases, as expected, as the number of observations decreases, but the estimates remain accurate down to a very small number of observations.

### 4.2 Digestion Curves

**FIGURE 4:**
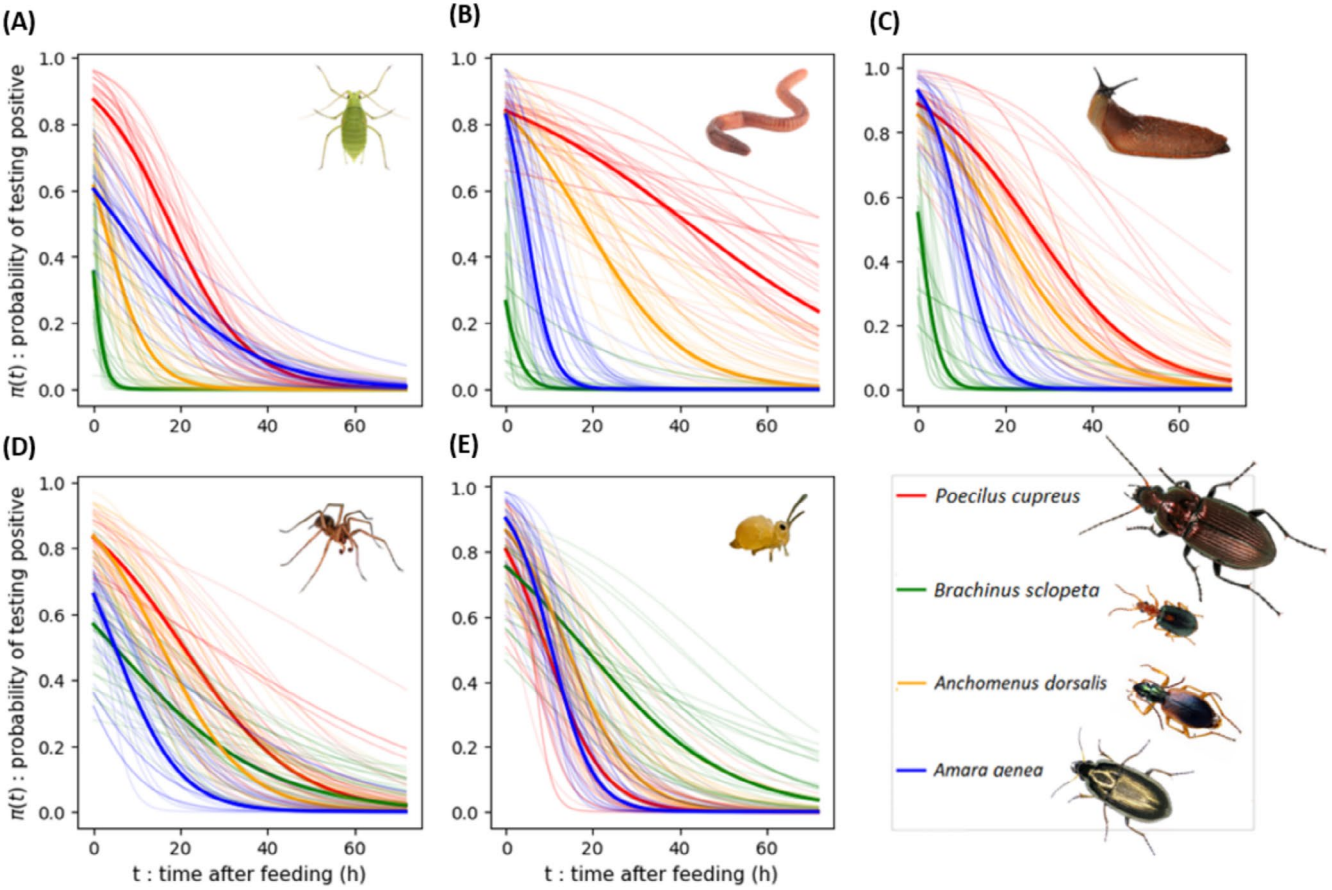
Posterior digestion curves of the five types of prey by the four most abundant carabid species in the dataset—On each panel, the thick bold line is the digestion curve obtained with $\beta_{0}^{p,c}$ and $\beta_{1}^{p,c}$ mean posterior values for each predator–prey pair and the 40 shaded lines a random set of posterior curves (i.e., the digestion curves for each predator–prey pair, produced by randomly choosing 40 pairs of $\beta_{0}^{p,c}$ and $\beta_{1}^{p,c}$ in the joint posterior distribution).

Figure 4 shows that digestion curves vary greatly among the 4 most abundant carabid species in the field and depend on the type of prey consumed. For example, a springtail meal is virtually undetectable 40 h after feeding in *P. cupreus* (D) while more than 72 h are required for an earthworm meal (C). Similarly, the digestibility of certain prey varies between carabid species. For example, the digestion curves of springtails by *A. aenea* , *P. cupreus* and *A. dorsalis* are very similar (D) while the digestion rate of earthworms or aphids (C & B) appears very contrasted between the four species. In particular, for earthworms (C), the digestion curves of *B. sclopeta* and *P. cupreus* differ radically. In *B. sclopeta*, the probability of detection is low from the early beginning and decreases rapidly to reach 0 after only 10 h, whereas in *P. cupreus* , the probability of detection is high immediately after feeding (almost 1) and decreases very slowly, still remaining over 0.2 72 h after ingestion. If we assume that bigger meals take more time to digest, then higher voracity can explain slower digestion rates for *P. cupreus* and *A. dorsalis* compared to *B. sclopeta* (see also Supporting Information S11). This example clearly illustrates the need to take digestion curves into account if predation rates are to be correctly estimated from molecular detection data.

### 4.3 Predation Rates

**FIGURE 5:**
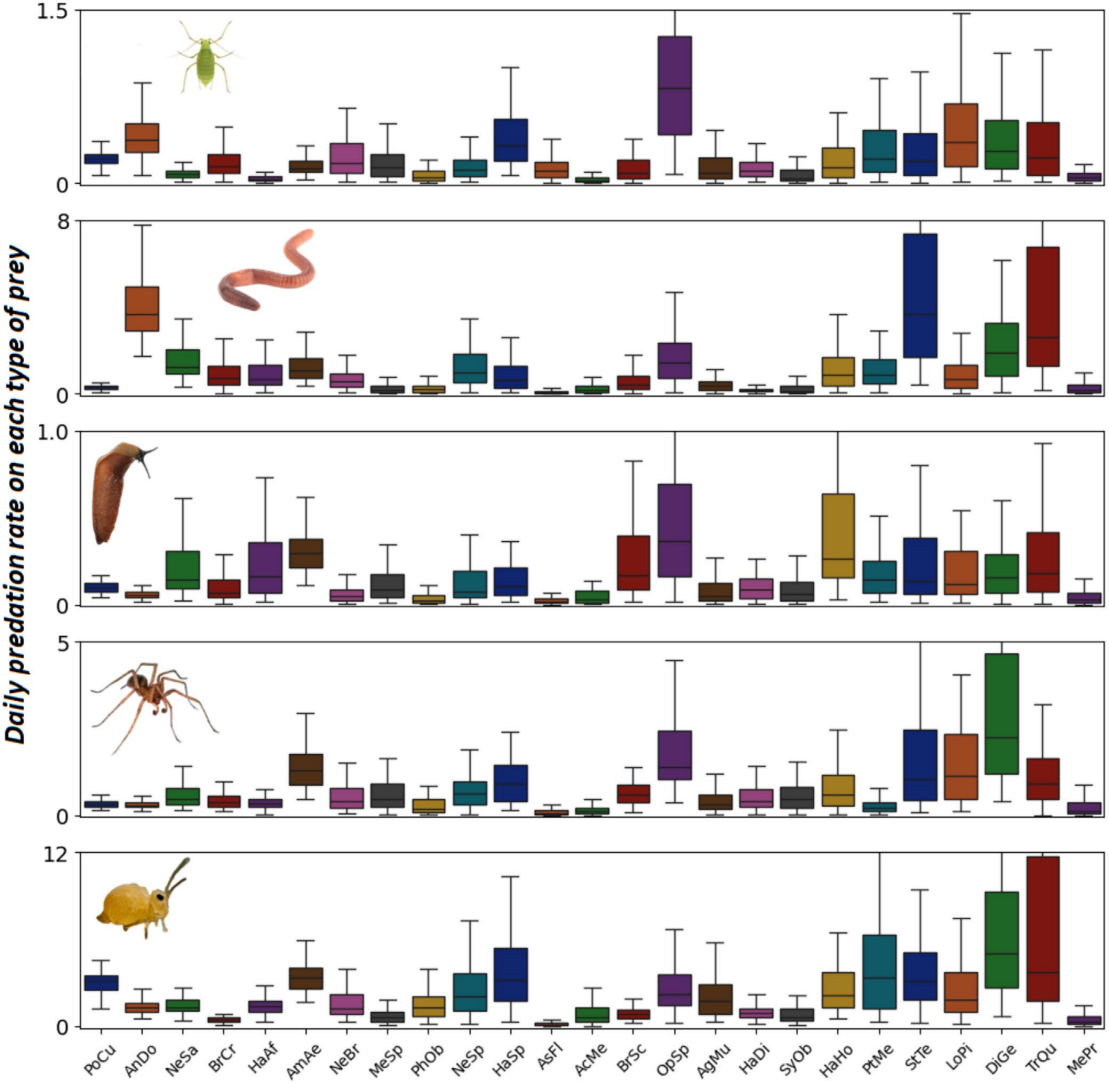
Daily predation rates—Boxplot (median and range) of predation rate estimates on each prey type (i.e., each panel) for each carabid species (*x*‐axis) present in the dataset (see Supporting Information S10 for carabid code names). The *y*‐axis range (number of predation events per day) is different for each prey type, as the order of magnitude of predation rate varies between prey types. Colours differ for each carabid species for visual clarity only, and the carabid species are ranked in descending order of abundance in the field. A visualisation of the full posterior distributions can be found in Supporting Information S8. Posterior distributions of some key prey, predator and interaction effects are also presented in Supporting Information S12. See also Supporting Information S6 for mean values and standard deviations of daily predation rates.

The estimates of predation rate provided by our model fall within a reasonable order of magnitude, both in their median value and in its variability among species. For example, the median predation rate on slugs varies from 0.02 feeding events per day for *Phyla obtusa* and *Asaphidion flavipes* to 0.29 for *A. aenea* and 0.36 *for Ophonus* sp. The maximum estimated rate of predation is of the order of 5 prey items consumed per day (median value for predation of springtails by *Diachromus germanus*). For all prey types, the median predation rate varies roughly by a factor of 10 among predators. This wide range of variation confirms that the biocontrol potential varies greatly according to the composition of the carabid community. Interestingly, distinct predator strategies emerge, with some species favouring predation on a limited number of prey types (e.g., *A. dorsalis* on earthworms and aphids), while many others are true generalists, some even showing high predation rates on all prey types (e.g., *Trechus quadristriatus* and *Stenolophus teutonus*). On the contrary, certain carabid beetles (e.g., *A. flavipes* ) appear to be infrequent consumers of the prey studied (Figure 5).

### 4.4 Community‐Scaled Biocontrol Indicator—Application to the Study of Seasonal Variation

**FIGURE 6:**
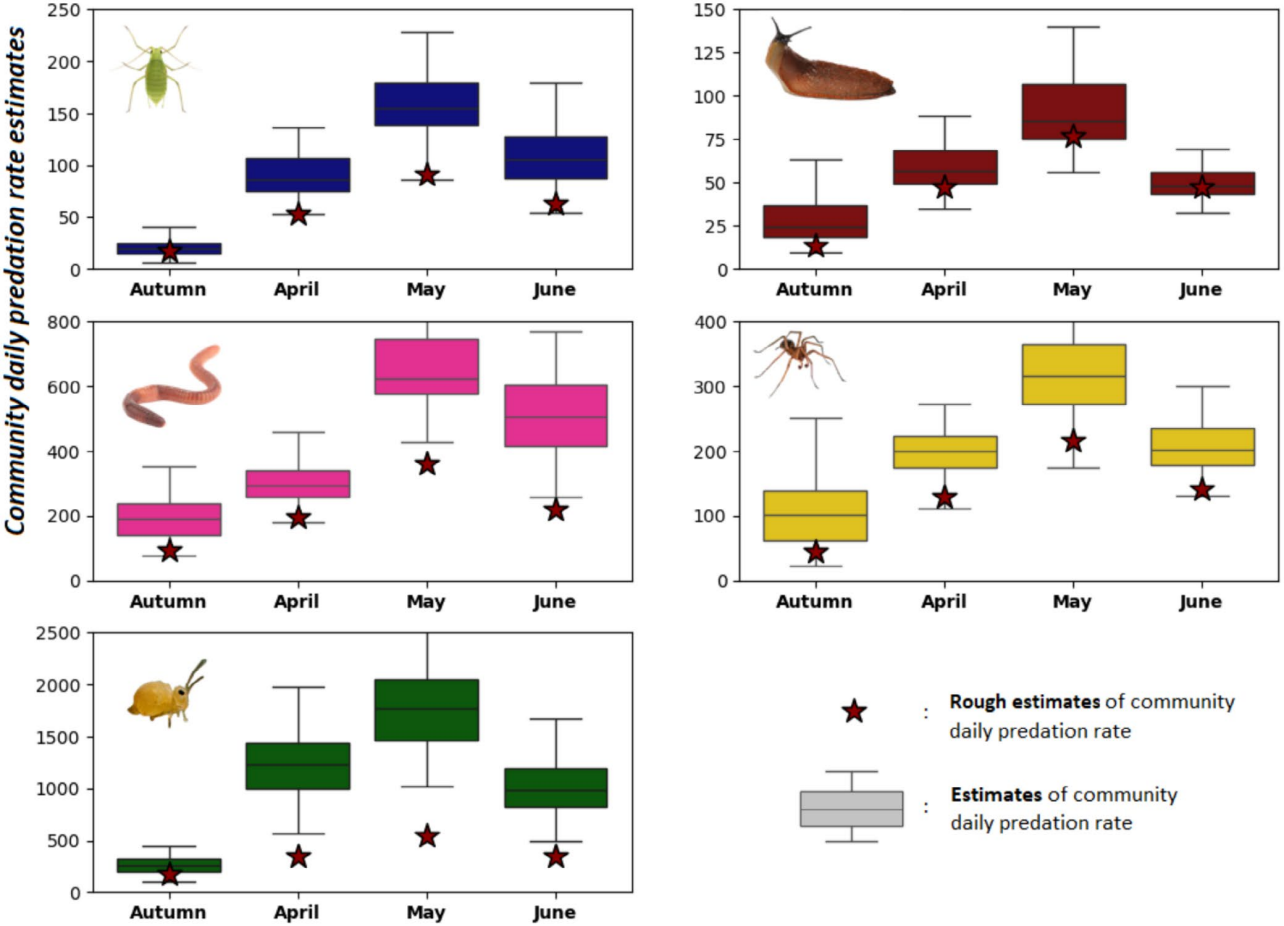
Changes in the biocontrol indicator over the cropping season for the five types of prey—Boxplots show the average predation rate (per day) exerted by the entire community of carabid beetles at each period (*x*‐axis) on each type of prey. The average predation rate (per day) was calculated according to Equation (6) (3.2). Star symbols represent rough estimates, that is, the sum of species abundances weighted by the detection frequency of each prey (see Section 3.2). Autumn refers to the months of November and December.

Unsurprisingly, the biocontrol indicator was mainly determined by activity‐density, reaching maximum values in May for all prey types. However, the differences in biocontrol indicator values between the autumn and spring communities were less marked for earthworms, slugs and spiders than for aphids and springtails. This result can be explained by a change in the composition of the carabid community, leading to a change in food preferences. The seasonal variations of our estimates were similar but more pronounced than those of the rough estimates (star symbols in Figure 6) based on species abundances weighted by the detection frequencies (see Section 3.2). However, the rough estimates systematically underestimated the actual predation rate. The magnitude of the underestimation depended strongly on the prey type considered: it was the lowest for slugs (rough estimates systematically falling between the 25th and 75th quantile of our estimates) and the highest for springtails (rough estimates 3 to 4 times lower than the median of our estimates and systematically below the 99th quantile). This discrepancy can be attributed to variations in digestion curves between prey types. Taking the most abundant *P. cupreus* as a reference species (see Figure 4), the difference between the rough prediction and our biocontrol indicator is greater the higher the digestibility of the prey.

## 5 Discussion

We have developed a new framework for estimating predation rates of inconspicuous predator species in complex assemblages of terrestrial predators and preys in the field. It is based on a hierarchical Bayesian model mobilising two sets of binary‐qualitative data based on the molecular detection of prey in predators (e.g., PCR results), one in individuals captured in the field, the other in individuals fed in the laboratory. Simultaneous processing of both datasets enabled us to model the gradual extinction of the molecular signal in predators resulting from prey digestion and to estimate prey predation rates accordingly. This new method makes it possible to estimate the effects of the identity of each prey and predator and their interaction on the predation rate. Furthermore, the model we propose can accommodate deviations from the steady state assumption, which was previously considered essential (Andow and Paula 2023). The method successfully provided estimates of predation rates for each predator–prey pair in an assemblage of 25 predator species feeding on 5 types of prey. Thanks to the information transfer enabled by the hierarchical structure of the model, the predation rate can be obtained even for predator–prey pairs that are very poorly informed by the data, at the cost of greater uncertainties. The ecological interpretation of the estimated values for the model's parameters and hyperparameters enhanced our understanding of the biological system.

### 5.1 Model Performance and Biological Relevance

The goodness‐of‐fit of the model to data was very good (Figure 3) but this does not absolutely guarantee the biological relevance of the estimated predation rates. In the absence of direct observations in the field, whose difficulty of access is the very reason for this methodological research, the only values with which to compare our results are those obtained by the methods proposed previously or by expert knowledge. The digestion curves obtained in our application example were comparable to the few curves found in the literature (Greenstone et al. 2010, 2014) and in particular to those obtained in a previous study on the same data (Sacco‐Martret de Préville et al. 2024). Our estimates of predation rates were also all within the same orders of magnitude as those obtained with previous methods (see Supporting Information S13). Additionally, rough estimates of daily predation rates based solely on detection rates systematically yielded lower values than those inferred by our model (Figure 6)—a result that is not unexpected, given that in our case, prey DNA detectability typically does not exceed one day.

Alternatively, the performance of our model can also be evaluated through simulation. In Supporting Information S1, we developed a simulator to generate synthetic field datasets. Applying our model to these simulated datasets, we showed that it produced no significant errors when the underlying predation process followed a homogeneous Poisson distribution. More importantly, it remained accurate even when predation events were not independent but moderately aggregated.

Interestingly, our results were consistent with published information on predator diet. For example, the highest predation rate on earthworms was obtained for a presumed earthworm specialist, *A. dorsalis* (Larochelle 1990). Similarly, *L. pilicornis* , known in the literature as a springtail specialist (Larochelle and Association des entomologistes amateurs du Québec 1990), showed a high predation rate on this prey. More generally, small carabid beetles; had higher predation rates than large ones. Within a narrow taxonomic group such as ground beetles, the predation rate could vary according to predator–prey body mass ratio (Rall et al. 2012). This result could therefore indicate that the size distribution of prey in the field, for prey revealed by molecular detection, favoured their predation by small carabid beetles. In contrast, the feeding preferences of the carabid beetle *A. aenea* , traditionally described as phytophagous (Kromp 1999), were not confirmed by this study, which focused on animal prey. Its high predation rates on spiders, slugs and springtails combined with good digestion capacities observed in the laboratory rather indicate an omnivorous behaviour in our field conditions.

### 5.2 Applicability and Conditions of Application

The applicability of our model is primarily limited by the ability to collect relevant molecular data on predation and digestion. Limitations arise from the fact that digestion may be affected by the simultaneous consumption of several prey items, and from variations in the predation rate depending on factors such as season or temperature. In addition, molecular methods present inherent limitations, such as the inability to distinguish between dead and live prey, or the stages of prey consumed (eggs, larvae, adults), which have been extensively discussed in the literature (King et al. 2008; Traugott et al. 2013; Furlong 2015). However, in agreement with the dozen models published on this topic since (Dempster 1960), we believe that these limitations are not substantial enough to prevent the application of the model to a wide range of predators, as well as certain phyto‐ or coprophagous insects (e.g., granivorous carabid beetles (Carbonne et al. 2020)).

Our method is applicable to all binary‐qualitative data, provided that (i) not all detection tests are positive and (ii) both laboratory and field data are obtained using the same molecular method and protocol. While PCR diagnostics are a simple and well‐established tool, our model can be combined with any of the molecular approaches outlined in paragraph 3 of the introduction. Moreover, our approach is primarily intended for systems in which prey species are already known. However, it could also be applied to presence/absence data obtained through metabarcoding in systems with unknown prey, either by excluding unidentified prey or by assuming that the DNA decay rates of these taxa are comparable to those studied under laboratory conditions (Uiterwaal and DeLong 2024). Finally, the model could be extended to other types of samples, such as faeces, provided slight adjustments are made—particularly to the detectability decay curve. For example, with faecal samples, detection may be delayed due to intestinal transit time, shifting the peak of detectability to several hours after ingestion. In such cases, a right‐skewed, long‐tailed decay function would be more appropriate than the logistic function currently used.

One of the main conceptual differences between available models concerns the way prey biomass is accounted for. Most models (including ours) adopt an implicit approach and rely on the quantity of prey ingested at the beginning of the feeding trial (= voracity). Such models typically incorporate voracity through their representation of molecular signal decay, for example using a half‐life value (Greenstone et al. 2010, 2014; Sacco‐Martret de Préville et al. 2024; Uiterwaal and DeLong 2024). In our model, voracity is defined as the maximum quantity of prey that a predator can contain, and we assume that this quantity is systematically reached after each predation event. Since the 1980s, other models—combined with quantitative molecular methods—have been based on the explicit monitoring of prey quantity (e.g., biomass or DNA concentration) within the predator (Sunderland et al. 1987; Andow and Paula 2023).

The recent model of (Andow and Paula 2023) uses the latter approach to estimate per capita predation rate from the average quantity of prey DNA in the predators and its rate of decay by digestion, which can be approximated by the inverse of its half‐life. The use of quantitative data makes it possible to obtain accurate results with few observations. However, the model relies on the assumption that the quantity of prey in predators is constant (steady state hypothesis, see also Section 5.4), which is questionable, especially when the predation rate is low. In contrast, our model only considers the probability of detection, which is better suited to binary‐qualitative data. When the proportion of negative tests is very low, our method reaches saturation and it becomes necessary to measure the amount of prey in the predator, as in (Andow and Paula 2023). However, as soon as the frequencies of positive and negative tests remain reasonably balanced and the uncertainties and biases associated with the molecular method used are the same for both datasets, our estimates are less sensitive to the molecular method used compared to direct measurements (Andow and Paula 2024).

Finally, the amount of data required to calibrate a model is also crucial to its applicability. Relying on its hierarchy to make the most of each piece of data, we have calibrated our model on a system of 5 prey types × 25 carabid species = 125 interactions, with 1534 observations in the field and 567 in the laboratory; that is < 20 observations per predation rate. In Supporting Information S5 and code we show that it is possible to calibrate the model with even less data.

### 5.3 Potential Uses in Biocontrol and Beyond

As highlighted in (Vialatte et al. 2021), there is an urgent need to quantify pest regulation, especially at the community level of generalist predators that prey on multiple pests. Indeed, there is strong evidence that the abundance and diversity of natural enemy communities promote natural regulation of pests (Vialatte et al. 2021). However, while the mechanisms involved are generally understood, we still lack the tools to measure them accurately. Some empirical approaches exist, such as the use of exclusion cages (Birkhofer et al. 2017), but these methods are often biased (Furlong 2015) and fail to disentangle the contributions of individual predator species within the community. To the best of our knowledge, our application example on carabid communities represents one of the most comprehensive attempts to quantify the predation rate exerted by an entire predator community on a set of key prey species.

In line with previous work on this issue (Feit et al. 2019; Perennes et al. 2023) we provide a framework that not only inherently considers several preys but provides predation rate estimates both at species and community scales (see Section 3.2). In this way, our model allows not only to estimate the predation rate exerted by the community, which can be translated into a quantification of ecosystem services or dis‐services depending on the prey consumed (e.g., pest or decomposer), but also to identify, for example, the species that contribute most. Combined with a reliable measure of predator density, which is another challenge (Ahmed et al. 2023), our model provides an unprecedented estimate of the quantity of pests consumed per unit area. Unlike all the proxies and indirect measures currently in use, this estimate would allow direct quantification of the effects of different control strategies (management practices or crop diversification) on pest regulation.

Finally, our model is flexible enough to be used to shed light on more fundamental ecological issues, especially the origins and consequences of predation rate variations. It could be used to analyse the effects of the environment (e.g., season, habitat, prey availability) or predator traits (e.g., size, morphology, diet) on predation rate, as we did with season in (see Section 4.4, Supporting Information S5 and Section 5.4 below). One way to do this is to recalibrate the model on subsets of the dataset, as we did in (Supporting Information S5 and code) for each sampling session. At community scale, our estimates provide valuable information concerning species interactions, such as competition for resources and prey switching, which would help us better understand niche differentiation and the determinants of community structure.

### 5.4 Future Directions

One simplification of our model, shared with all existing models in the literature, is that predation and digestion rates are constant (steady state hypothesis). This is obviously not true. However, our model is flexible enough to account for various biotic and abiotic factors that have a proven influence on predation or digestion rates, either by adjusting the hierarchical structure of parameters or more fundamentally by introducing new variables to overcome the steady state hypothesis.

A first modification could involve replacing the taxonomic hierarchy used in our study with one based on predator traits (such as size, diet or reproductive season), which are available for several taxa. A second improvement to the model could focus on parameter decomposition (see Section 2.4); for instance, by explicitly incorporating the effects of season or prey availability. Indeed, prey availability strongly influences predation rates, and in this study, low predation rates on aphids have likely resulted from low aphid densities. Although not directly measured here, previous research has shown mixed effects of prey availability on predation, with some studies finding increased predation at higher prey densities in laboratory conditions (Rothschild 1966; Ge et al. 2019), while others in microcosms, like (Naranjo and Hagler 2001), reported a low impact except at very low densities. In our case, prey density was indirectly considered using the predator's season of capture as a proxy. In the future, it would be feasible to directly integrate the effect of season or prey density levels into the decomposition of the predation rate (see Section 2.4).

More substantial modifications to the model could also be considered, particularly to overcome the steady state hypothesis. (Andow and Paula 2023) proposed a rough linear adjustment of their estimates in cases where predation is not in steady state, based on the increase or decrease of prey biomass divided by the time elapsed between two measurements. We believe that our model is better suited to modifications designed to represent non‐stationary situations. Regarding digestion, and since voracity can be measured as part of the feeding experiment (Sacco‐Martret de Préville et al. 2024), it is conceivable to integrate it explicitly into the digestion equation. For predation, it is also simple to introduce a variable predation rate, for example, by making it dependent on temperature or the day/night cycle. These improvements would introduce new sources of variability in detection rates, and each must be tested to ensure they do not prevent the model's calibration. As an example, in (Supporting Information S14), we developed a second version of the model that allows the predation rate to vary depending on the (random) moment when the carabid falls into the pitfall trap, and we show that the model's calibration remains robust with this modification.

In conclusion, our model offers a powerful new lens to explore and quantify predation in complex ecological systems where direct observation is almost impossible. By leveraging relatively simple binary molecular data, it enables the estimation of daily predation rates and reveals how these rates vary with predator identity, prey type and seasonal community dynamics. Thanks to its hierarchical structure, the model can produce estimates even for rare predatory species or poorly documented predator–prey pairs, while appropriately reflecting the associated uncertainty. Applied at both species and community scales, it highlights functional differences among predators and provides a comprehensive ecological indicator for evaluating biocontrol potential. Beyond biocontrol applications, it offers a flexible framework to investigate broader ecological issues related to trophic interactions and ecosystem functioning.

## Author Contributions

Conceptualization, A.M., M.‐P.E. and M.P.; formal analysis, A.M., K.R. and M.P.; data collection, A.S.‐M.P., E.C., M.P.; writing – original draft preparation, A.M., Y.T., S.P. and M.P.; writing – review and editing, A.M., K.R., M.‐P.E., A.S.‐M.P., E.C., Y.T., S.P. and M.P.; supervision, M.P., Y.T. and S.P.; project administration, Y.T., M.P.; funding acquisition, Y.T., E.C. and M.P. All authors have read and agreed to the published version of the manuscript.

## Disclosure

Benefit sharing statement: Benefits from this research accrue from the sharing of our codes and results on public databases as described above.

## Conflicts of Interest

The authors declare no conflicts of interest.

## Supporting information


**Data S1:** men70033‐sup‐0001‐DataS1.pdf.

**Data S2–S13:** men70033‐sup‐0002‐DataS2‐S13.docx.

## Data Availability

Both field (https://doi.org/10.57745/BBHYYX) and feeding trial (https://doi.org/10.57745/XXV1XQ) data are available on the Recherche Data Gouv Repository. Codes are available on this repository: https://zenodo.org/records/14412050.
