# Supplementary material for: Unveiling the Hidden Feast: A Model to Translate Molecular Detection Into Predation Rate—Application Example on Biological Control by Generalist Predators in Agricultural Fields
Source: Mol Ecol Resour. 2025 Aug 20;25(8):e70033. doi: 10.1111/1755-0998.70033 (PMC12550487; doi:10.1111/1755-0998.70033)
Supplement: Supplementary file 1 — Data S1: men70033‐sup‐0001‐DataS1.pdf. [file MEN-25-e70033-s001.pdf]

Supplementary Material 1: Aggregated Predation Events

This script complements the method for estimating predation rates from molecular data collected in the field and in the laboratory, as described by [Masson et al. 2024]. It outlines a protocol for simulating realistic predation behaviors and, based on these, generating synthetic molecular datasets analogous to those obtained in field and lab conditions. These simulated data are then used to calibrate our model and assess the robustness of the resulting estimates under more complex predation scenarios.

In the model proposed by [Masson et al. 2024], predation was initially represented as a **homogeneous Poisson process**, assuming that **predation events are independent** from one another and occur at a constant rate. This choice was made for the sake of simplicity and because the predation behavior of carabid beetles in our study system remains largely unknown. However, the homogeneous Poisson process describes a rather specific behavior, akin to a random walk, which likely does not reflect the actual behavior of predators in the field. For most predators, it is more realistic to assume that predation events are aggregated in time, rather than uniformly distributed.

There are several ways to simulate temporally aggregated predation events. One approach is to use an **inhomogeneous Poisson process**, where the predation rate varies over time according to a deterministic function—such as a day/night cycle or a sigmoidal curve to model diurnal or nocturnal hunting activity. This type of model introduces temporal structure without directly linking the events themselves. A more general approach, particularly relevant when prey are themselves aggregated in space or time, is to introduce an explicit dependency between events. For example, in a **self-exciting process** such as the **Hawkes** process, the dependency between events is expressed as a temporary excitation following each new event, which increases the short-term likelihood of further events and naturally leads to their aggregation in time. This is the approach we adopted to build the simulator presented here.

1. Importing Data and Useful Functions

In [6]:
```
import matplotlib.pyplot as plt

import pandas as pd
import numpy as np
import math
import os

from scipy.integrate import quad
from scipy.stats import truncnorm

from Hawkes_Utils import simulate_hawkes
from Hawkes_Utils import build_simulated_dataset, build_simulated_digestion_data
from Hawkes_Utils import plot_hawkes_simulation, plot_hawkes_intensities, plot_l
```

```
# Set working directory (adjust to your local environment)
os.chdir('C:/Users/abmasson/Desktop/Hawkes')

# Ensure reproducibility
np.random.seed(43)

# Load predation estimates from MER model
df_EstimatesMER = pd.read_excel("./Data/Estimates_MER.xlsx")
df_EstimatesMER = df_EstimatesMER[df_EstimatesMER.columns[1:]]
```

2. Simulator Definition

The simulator defined below is intended to reproduce the field data input used in the model by [Masson et al. 2024], i.e., to simulate PCR tests detecting prey DNA in predators captured in the field. More precisely, the simulation proceeds in three steps:

(2.1) simulate the **predation process**, by generating a list of aggregated predation events over a time period T, following a Hawkes process; **(2.2)** simulate the **digestion process**, by modeling the decreasing detectability of each event as a function of the time elapsed between the predation event and the test conducted at t=T; **(2.3)** simulate the **observation process**, i.e., generate the partial information available under real-world conditions: the result of the PCR test performed at t=T, which indicates whether at least one predation event was detected.

2.1 Predation Process – Hawkes Process

The **Hawkes process** is a **point process** whose intensity at time t depends on past events (this is referred to as the **conditional intensity**). The version we use in this script has the following conditional intensity function at time t:

$$\lambda(t) = \mu + \sum_{t_i < t} \alpha \cdot e^{-\beta(t - t_i)}$$

where:

- $\lambda(t)$: intensity at time t
- $\mu > 0$: baseline (constant) intensity
- $\alpha > 0$: excitation strength of each event
- $\beta > 0$: exponential decay rate
- t_i: times of past events, such that $t_i < t$

Notes:

- Each new event causes an **excitation**, i.e., a temporary increase in the process intensity by an amount α. This excitation decays exponentially over time until the intensity returns to the baseline μ.
- The **homogeneous Poisson process** used in our original model corresponds to the Hawkes process with **no aggregation** of predation events, that is, $\alpha = 0$.

- The **expected number of offspring events** generated by a single event is given by $\eta = \frac{\alpha}{\beta}$. To ensure that the process remains stable (i.e., does not explode), the condition $\alpha < \beta$ must be satisfied. In the remainder of this document, we interpret η as a **predation event aggregation rate**.
- The **expected intensity** of the Hawkes process has the analytical expression:

$$\mathbb{E}[\lambda(t)] = \frac{\mu}{1 - \eta}$$

2.2 Digestion Process

The digestion process is identical to the one used in the method described by [Masson et al. 2024]. The detectability curve of a predation event is still denoted by π. In this simulator, we do not vary the values of its parameters β_0 and β_1.

$$\text{logit}(\pi(t)) = \beta_0 - \beta_1 \cdot t$$

Notes:

- It is possible that the **aggregation of predation events** also affects their detectability. For example, if each predation event resets the detectability of the prey to its maximum value, a new predation event could **mask** previous ones — especially if the events are aggregated. Conversely, a **cluster of aggregated predation events** may remain detectable for a longer period. Since our model estimates the detectability of each event **independently of others**, it does not account for any potential influence of aggregation on detectability. Moreover, these considerations relate to the broader question of **whether or not prey biomass should be accounted for**, which we do not address in this appendix.

- *[Technical]* In the code, because the model was calibrated this way, we use the equivalent expression:

$$\pi(t) = \frac{\exp(\beta_0 + \beta_1 \cdot t)}{1 + \exp(\beta_0 + \beta_1 \cdot t)}$$

  with $\beta_1 < 0$.

2.3 Observation Process

The goal of this process is to simulate the result of the PCR test performed on the stomach contents of field-collected predators at time $t = T$. To do so, we simulate the detection (or non-detection) of each predation event **independently**, by drawing from a Bernoulli distribution with parameter $p = \pi(T - t_i)$, where $T - t_i$ is the time elapsed between the predation event and the sampling. If **at least one** event is detected, the PCR test result is considered **positive**; otherwise, it is **negative**.

Notes:

- Since the **excitation** caused by a predation event is independent from the **decay in detectability**, our process does **not** fall under the category of **marked Hawkes processes**. Straying from the usual frameworks, we will not attempt to derive or use **analytical expressions**, such as the expected value of the process. All values — particularly the **mean intensity** λ — will be computed via **simulation only**.

2.4 Simulation Function

This function is shown here solely to illustrate the implementation of the simulator. However, the actual function used is `simulate_hawkes`, as defined in `Hawkes_Utils`. **Do not uncomment.**

def simulate_hawkes(mu, alpha, beta, T, beta0, beta1, dt, seed=None, shortcut=False): """ Simulates a Hawkes process with logistic decay in detectability. Special case: if alpha = 0, the process is a homogeneous Poisson process with rate mu. """ if seed is not None: np.random.seed(seed) lambda_times = np.arange(0, T + dt, dt) lambda_values = [] # Special case: homogeneous Poisson process if alpha == 0: n_events = np.random.poisson(mu * T) events_latent = np.sort(np.random.uniform(0, T, size=n_events)) # constant intensity lambda(t) lambda_values = [mu] * len(lambda_times) else: # Hawkes process events_latent = [] t = 0 lambda_max = mu while t < T: u = np.random.uniform() w = -np.log(u) / lambda_max t += w if t > T: break lambda_t = mu + np.sum(alpha * np.exp(-beta * (t - np.array(events_latent)))) d = np.random.uniform() if d <= lambda_t / lambda_max: events_latent.append(t) lambda_max = lambda_t else: lambda_max = lambda_t events_latent = np.array(events_latent) for t_eval in lambda_times: history = events_latent[events_latent < t_eval] lambda_t = mu + np.sum(alpha * np.exp(-beta * (t_eval - history))) lambda_values.append(lambda_t) # Detection of observed events pi = 1 / (1 + np.exp(-(beta0 + beta1 * (T - events_latent)))) detected = np.random.uniform(size=len(events_latent)) < pi events_observed = events_latent[detected] stochastic_lambda_value = len(events_latent) / T if shortcut: Detection_pcr = int(len(events_observed) != 0) return Detection_pcr, stochastic_lambda_value return events_latent, events_observed, lambda_times, lambda_values, stochastic_lambda_value

3. Parameterization & Simulation

In the following chunk, we provide a simple example of the simulation. To parameterize it, we select a specific prey–predator pair (`predator` and `prey` in the chunk below). The parameters β_0, β_1, and μ are derived from the model estimates for this pair. The parameters α and η, as well as the simulation duration (T) and time step (dt), must be defined at the end of the chunk.

Choice of prey–predator pair

In [20]:
```python
predator = 'LoPi' ; prey = 'springtail'
```

List of predators:

In [22]:
```python
pd.unique(df_EstimatesMER.Predator)
```

Out[22]:
```
array(['AcMe', 'AgMu', 'AmAe', 'AnDo', 'AsFl', 'BrCr', 'BrSc', 'DiGe',
       'HaAf', 'HaDi', 'HaHo', 'HaSp', 'LoPi', 'MeSp', 'NeBr', 'NeSa',
       'NeSp', 'OpSp', 'PhOb', 'PoCu', 'PtMe', 'StTe', 'SyOb'],
      dtype=object)
```

List of prey types:

In [24]:
```python
pd.unique(df_EstimatesMER.Prey)
```

Out[24]:
```
array(['aphid', 'earthworm', 'slug', 'spider', 'springtail'], dtype=object)
```

Parameterization

In [26]:
```
# Initial parameters derived from the calibration of our method
df_param = df_EstimatesMER[(df_EstimatesMER['Predator'] == predator) & (df_Estim

beta0, beta1 = df_param['beta0_pc'].mean(), df_param['beta1_pc'].mean()
mu = df_param['hourly_lambda_pc'].mean()

# User-defined parameters for the Hawkes process
T = 50 ; dt = 0.1  # simulation duration and time step
eta = 0.5 ; alpha = 0.5 ; beta = alpha / eta  # Hawkes process parameters
```

In [27]:
```
events = simulate_hawkes(mu, eta, alpha, beta, T, beta0, beta1, dt, seed=None, s
```

Simulation display

In [29]:
```
plot_hawkes_simulation(events, T=T)
```

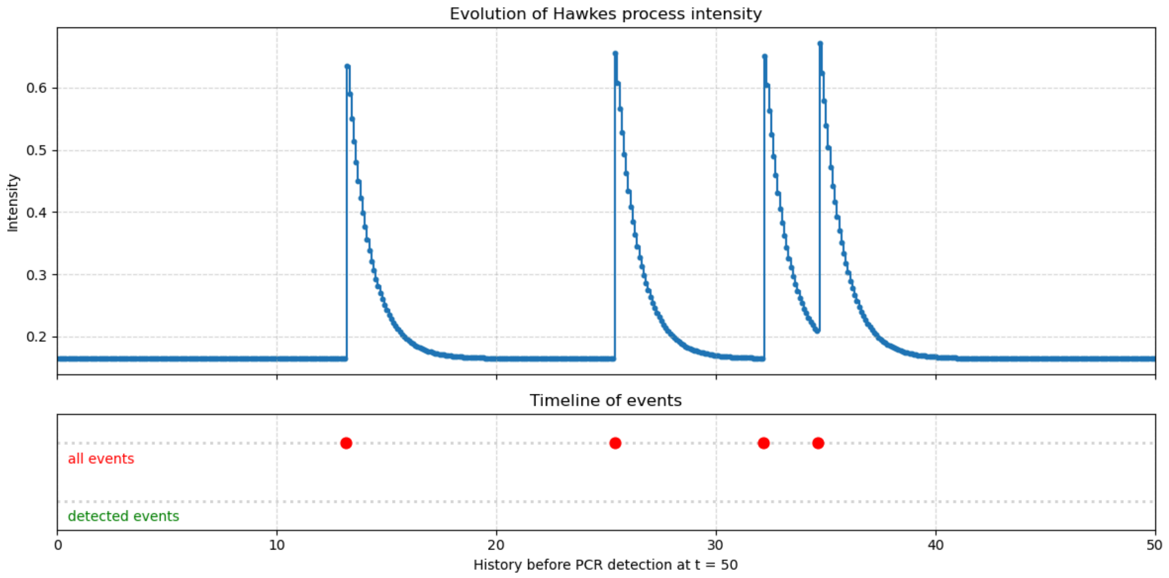

Figure 1: Evolution of the Hawkes process intensity and timeline of predation events — events that occurred (in red) and those that were detected (in green). The x-axis is shared between both plots and represents the time preceding the PCR detection at $t = 50$. On the right ($t = 0$) are the oldest events; on the left ($t = 50$) are the most recent ones.Since the process is stochastic, the figure may vary each time the script is run.

4. Simulation of Datasets

The goal of this section is to build datasets that serve as **simulated analogues** of the field data, but with different underlying values of **predation event aggregation** (assimilated to the expected number of offspring per event η). We generate several datasets, one for each level of predation event aggregation (each value of η). As mentioned in the introduction, η varies between 0 and 1, and we choose to explore its full range by considering the following values: **[0.0, 0.25, 0.5, 0.75, 0.9]**.

The key challenge here is to ensure that simulations are properly parameterized. To increase the relevance of our evaluation, we use fixed values that are representative of the field system. Specifically, we set the digestion curve parameters β_0 and β_1 to the **mean of their posterior distributions**.

To ensure that **all simulations share the same expected number of predation events** λ, regardless of the aggregation level, we exploit the analytical expectation of a Hawkes process, $\mathbb{E}[\lambda] = \frac{\mu}{1-\eta}$ and assign the **baseline intensity** of every simulation to $\mu = \lambda.(1-\eta)$. Finally, after a graphical analysis of the simulations (see Figure above) we decided to **fix** the excitation strength to $\alpha = 0.5$, and derive the corresponding decay rate $\beta = \alpha/\eta$ for each value of η.

4.1 Example: Simulation with *Loricera Pilicornis* preying on springtails

To get a sense of how predation intensity varies across different levels of prey aggregation (i.e., different values of η), we can run a simulation with a selected predator and prey type (defined in the cell and lists below). By default, we use *Loricera Pilicornis* preying on springtails (*LoPi* is known to be a specialist of springtails). The results of these simulations are shown in **Figure 2** below.

In [35]:
```
predator = 'LoPi' ; prey = 'springtail'
```

Parameterization

In [37]:
```
all_events = []
df_param = df_EstimatesMER[(df_EstimatesMER['Predator'] == predator) & (df_Estim

# Fixed parameters derived from model calibration
beta0 = df_param['beta0_pc'].mean()
beta1 = df_param['beta1_pc'].mean()
lambda_Mod = df_param['hourly_lambda_pc'].mean()

# User-defined Hawkes process parameters
eta_sets = [0.25, 0.5, 0.75, 0.9]

T = 50 ; dt = 0.1

for eta in eta_sets:

    if eta == 0:
        alpha = 0
        beta = 0
    else:
        alpha = 0.5
        beta = alpha / eta

    mu = lambda_Mod * (1 - eta)
    events = simulate_hawkes(mu, eta, alpha, beta, T, beta0, beta1, dt,
                             seed=None, shortcut=False)
    all_events.append(events)

plot_hawkes_intensities(all_events, T, eta_sets, lambda_Mod)
```

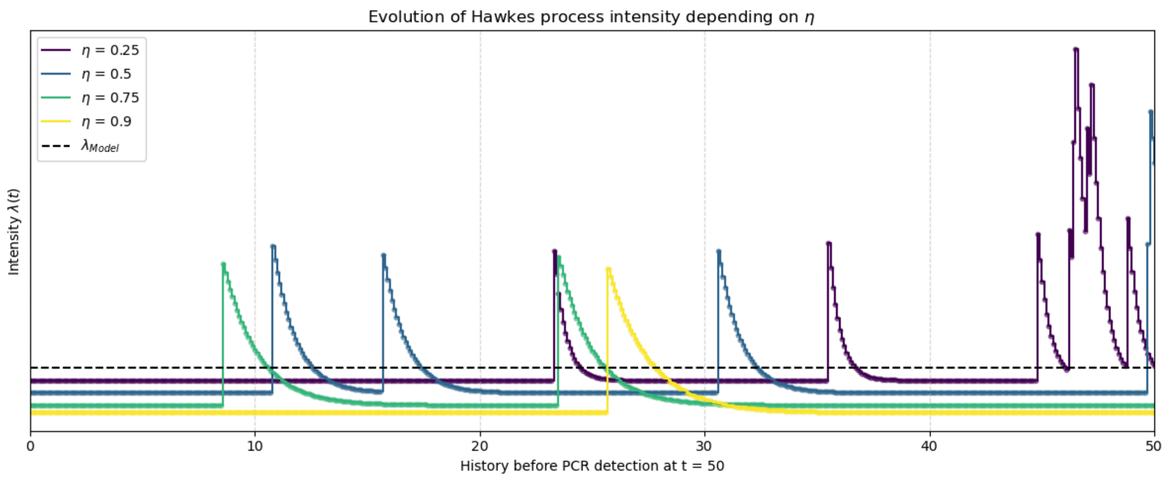

Figure 2: Evolution of Hawkes process intensity for different aggregation levels η. λ_{mod} corresponds to the predation rate estimated by our model, and serves as the **baseline intensity** μ for all simulated Hawkes processes. The dashed line represents the intensity of the **homogeneous Poisson process** used in our model. The other curves show the evolution of the Hawkes process intensity for the different values of η (see legend). Since the process is stochastic, the figure may vary each time the script is run.

4.2 Simulation of Detection Datasets

The datasets are simulated using the function `build_simulated_dataset()` and saved in the `"./Data"` folder under the following name: `f"Vanilla_field_data_eta_{str(eta_val).replace('.', '_')}.csv"`

Parameterization and simulation launch

In [42]:
```
# --- Parameters
T = 300
dt = 1
n_replicates = 300
eta_sets = [0.0, 0.25, 0.5, 0.75, 0.9]
save_path = "./Data/"
save_name = "Simulated_df_pred_eta_{}.csv"
save_digestion_name = "Simulated_df_dig.csv"

try:
    print("[INFO] Attempting to load existing simulation files...")
    str_eta = str(eta).replace('.', '_')
    df_field_simulated = pd.concat([
        pd.read_csv(os.path.join(save_path, save_name.format(str_eta)))
        for eta in eta_sets
    ], ignore_index=True)
    df_abaque = None

    df_dig_simulated = pd.read_csv(os.path.join(save_path, save_digestion_name))

    print("[INFO] Load successful: no simulation needed.")

except Exception as e:
    print(f"[INFO] Load failed ({type(e).__name__}) → launching simulations.")
    df_field_simulated = build_simulated_dataset(
        df_data=df_EstimatesMER,
```

```
        T=T,
        dt=dt,
        n_replicates=n_replicates,
        eta_sets=eta_sets,
        save=True,
        save_name=save_name,
        save_path=save_path
    )

    df_dig_simulated = build_simulated_digestion_dataset(
        df_data=df_EstimatesMER,
        output_path=os.path.join(save_path, save_digestion_name)
    )

    print("[INFO] Simulation completed and files saved.")
```

```
[INFO] Attempting to load existing simulation files...
[INFO] Load successful: no simulation needed.
```

5. Robustness Analysis of the Method

In this section, we aim to calibrate the model on the simulated datasets and compare the **predation rate estimates** it provides with the known λ values used in the simulations.

This step requires reusing the main script provided in the code associated with [Masson et al. 2024]. We do not reproduce the full procedure here, but the posterior samples resulting from model calibration on each dataset (for the 5 values of η) are available in the `./Samples` folder.

5.1 Inference of Predation Rate Estimates

The idea is to re-run the *Main Script.py* using the five simulated datasets corresponding to the five values of η — these are the `Simulated_df_pred` files stored in the `./Data` folder (e.g. `Simulated_df_pred_eta_0_0.csv`). To do this, the function `build_samples_and_estimates()`, defined in `Hawkes_Utils`, should be copied and pasted into `Main Script.ipynb`.

This function generates both the raw posterior samples (`Samples`) stored in the `./Samples` folder (e.g. `Samples_eta_00.nc`) and formatted parameter estimates (`Estimates`) for key model outputs, saved as Excel files (e.g. `Estimates_eta_00.xlsx`) in the `./Data` folder.

5.2. Summary Table for Analysis

Here we build `summary` dataframe, which contains all the λ values to be compared. For more details on the structure and contents of `summary`, see the definition of `build_extended_summary()` in `Hawkes_Utils.py`.

In particular, Each row in the summary corresponds to either an **estimate** or a simulated **observation**. In the case of an estimate, λ is a draw from the **posterior distribution** of the predation rate, as estimated by our model. In the case of an observation, λ is the

empirical mean predation rate computed during each simulation (i.e., the number of simulated events divided by the simulation duration).

In [49]:
```
summary = build_summary(df_EstimatesMER, eta_values=[0.0, 0.25, 0.5, 0.75, 0.9],
                        data_folder="./Data/",
                        estimates_path='Estimates_eta_{}.xlsx',
                        df_field_path='Simulated_df_pred_eta_{}.csv')
```

In [50]:
```
summary
```

Out[50]:

	Predator	Prey	Pair	lambda	Source	eta
**0**	AcMe	aphid	AcMe_aphid	0.024826	estimation	0.0
**1**	AcMe	aphid	AcMe_aphid	0.019430	estimation	0.0
**2**	AcMe	aphid	AcMe_aphid	0.014786	estimation	0.0
**3**	AcMe	aphid	AcMe_aphid	0.017761	estimation	0.0
**4**	AcMe	aphid	AcMe_aphid	0.018607	estimation	0.0
**...**	...	...	...	...	...	...
**229995**	SyOb	springtail	SyOb_springtail	0.000000	observation	0.9
**229996**	SyOb	springtail	SyOb_springtail	0.003333	observation	0.9
**229997**	SyOb	springtail	SyOb_springtail	0.003333	observation	0.9
**229998**	SyOb	springtail	SyOb_springtail	0.003333	observation	0.9
**229999**	SyOb	springtail	SyOb_springtail	0.006667	observation	0.9

230000 rows × 6 columns

5.3. Results of Robustness Analysis

In this section, we present two figures to compare the simulated and model-estimated values of λ. It is possible to change the prey and predator displayed in **Figure 3** using the next cell. By default, we focus on the consumption of springtails by *Loricera Pilicornis*.

In [53]:
```
predator = 'LoPi' ; prey = 'springtail'
```

In [54]:
```
pd.unique(df_EstimatesMER.Predator)
```

Out[54]:
```
array(['AcMe', 'AgMu', 'AmAe', 'AnDo', 'AsFl', 'BrCr', 'BrSc', 'DiGe',
       'HaAf', 'HaDi', 'HaHo', 'HaSp', 'LoPi', 'MeSp', 'NeBr', 'NeSa',
       'NeSp', 'OpSp', 'PhOb', 'PoCu', 'PtMe', 'StTe', 'SyOb'],
      dtype=object)
```

In [55]:
```
pd.unique(df_EstimatesMER.Prey)
```

Out[55]:
```
array(['aphid', 'earthworm', 'slug', 'spider', 'springtail'], dtype=object)
```

In [56]:
```
plot_lambda_distribution(summary, prey=prey, predator=predator)
```

```
C:\Users\abmasson\Desktop\Hawkes\Hawkes_Utils.py:463: FutureWarning: Use "auto" t
o set automatic grayscale colors. From v0.14.0, "gray" will default to matplotli
b's definition.
  sns.stripplot(
```

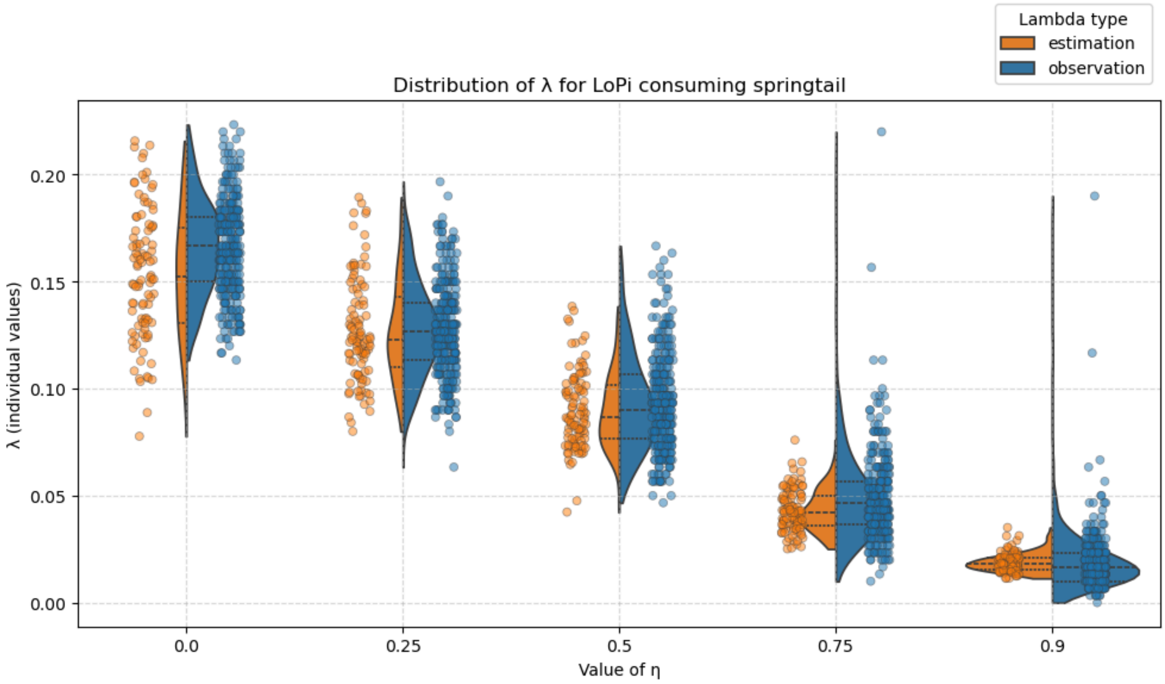

Figure 3: Violin plot of the predation rate λ — simulated values (right, in blue) vs. estimated values (left, in orange) — as a function of the aggregation level η. The orange distribution represents the posterior distribution of λ as estimated by our model (each dot corresponds to a posterior draw). The blue distribution corresponds to the distribution of empirical mean predation rates computed for each simulation (blue dots).

In **Figure 3**, we observe that in the case of springtail predation by *Loricera pilicornis*, the distributions of simulated values and the estimated posteriors of λ are quite close. More generally, for the vast majority of predator–prey pairs (see **Figure 4** below), the **mean simulated** λ systematically falls within the **75% confidence interval** of the estimated (posterior) distribution.

Although the simulation protocol was designed to produce datasets with the **same expected value of** λ, we observe that the **average empirical simulated predation rate** (i.e., the mean of means) tends to **decrease with increasing values of** η. This discrepancy is probably due to the fact that the analytical expectation $\mathbb{E}[\lambda] = \mu/(1-\eta)$ holds only in the **infinite time limit**, whereas our simulations are run over a finite duration ($T = 100$ h). Nonetheless, this does not prevent a meaningful comparison between simulated and estimated values.

For high aggregation levels ($\eta = 0.75$ or 0.9), we often observe a cluster of simulations (blue dots) with moderate numbers of predation events, alongside a few simulations with **very high empirical** λ, suggesting that the process has "taken off." These **extreme cases** tend to drive up the theoretical expectation $\mathbb{E}[\lambda]$ compared to the average empirical values actually observed.

Interestingly, the **simulated λ values become more dispersed** at higher η levels (especially $\eta > 0.5$), while the **posterior distributions** estimated by the model tend to **narrow**. This reflects the fact that the **Hawkes process introduces more variability** in the number of predation events than the **homogeneous Poisson process** assumed by our model.

In practice, the way we introduced temporal dependence between predation events does **not substantially impact the model's ability to estimate the true predation rate**.

In [59]:
```
plot_error_distribution(summary, prey="springtail", error_type = "relative")
```

```
C:\Users\abmasson\Desktop\Hawkes\Hawkes_Utils.py:530: FutureWarning: Use "auto" t
o set automatic grayscale colors. From v0.14.0, "gray" will default to matplotli
b's definition.
  sns.stripplot(
```

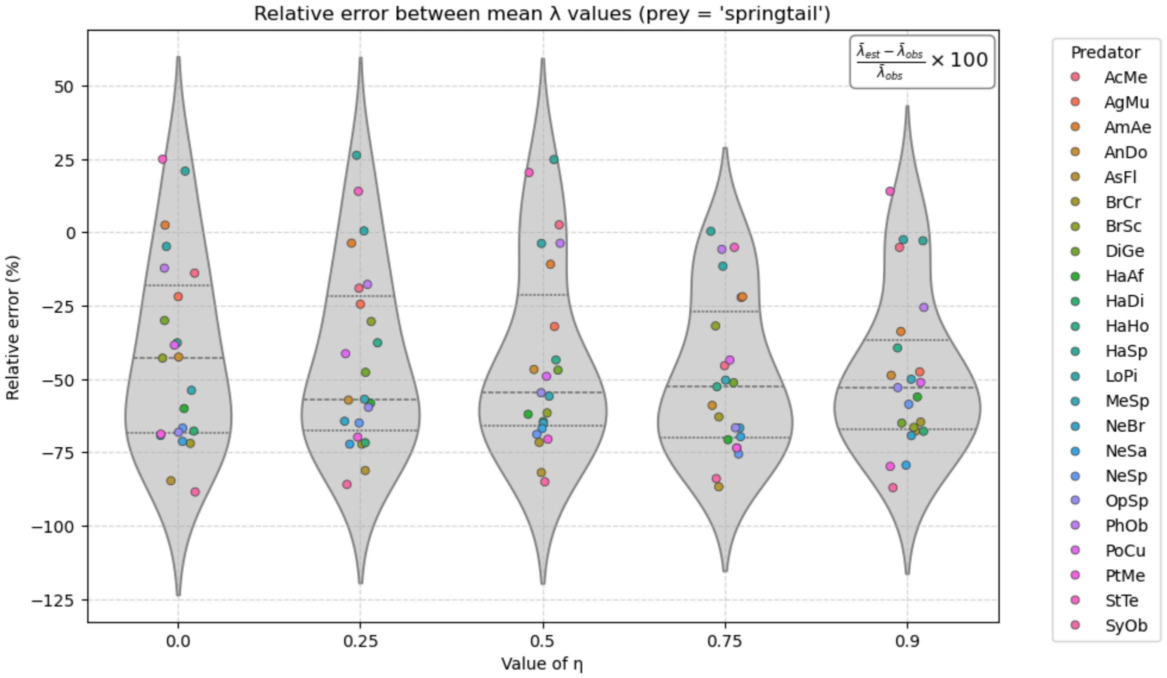

Figure 4: Violin plot of the relative error between the mean estimated predation rate and the mean simulated predation rate. The relative error is expressed as a percentage — negative values indicate that the model **underestimates λ**.

In **Figure 4**, we observe that the variability associated with the model's estimates is quite high, which partially obscures the effect of predation event aggregation. This result could likely be improved by using **higher-quality posterior samples**.

Overall, the **mean estimates** across all prey–predator pairs remain in the **same order of magnitude** as the simulated means (within approximately ±100). Given the inherent difficulty of the problem and the high variability typical of both ecological and molecular data, we consider this level of agreement to be satisfactory.

Finally we note that our estimates for **springtails** tend to **underestimate the true predation rate**, even when the simulated process is a **homogeneous Poisson process** — with an average underestimation of around **50%**. This bias does **not** appear in the

estimates for **aphids** or **earthworms**, for instance, and therefore remains difficult to interpret.

6. Conclusion

In this appendix, we explored the robustness of our predation rate estimation method [Massn et al. 2024] when applied to datasets generated under increasingly realistic conditions, namely with **temporal aggregation of predation events**. To this end, we replaced the homogeneous Poisson process used in our original model with a **self-exciting Hawkes process**, allowing us to simulate aggregated predation patterns.

Our analyses show that, overall, the model remains capable of producing **reasonable estimates** of the true predation rate, even under high aggregation levels. In most cases, the **mean of the posterior estimates** closely aligns with the **empirical mean predation rates** from the simulations. These results are particularly encouraging given the inherent noise and complexity of ecological and molecular data.

Watermark

In [65]:
```
%load_ext watermark
%watermark -n -u -v -iv -w -p pytensor
```

```
Last updated: Fri May 16 2025

Python implementation: CPython
Python version       : 3.11.8
IPython version      : 8.22.2

pytensor: 2.12.3

numpy     : 1.25.2
pandas    : 2.2.1
json      : 2.0.9
matplotlib: 3.8.3

Watermark: 2.4.3
```

■ *End of document*
